# Preadmission CHA_2_DS_2_-VASc Scores on Diastolic Function and Functional Outcome After Stroke with Nonvalvular Atrial Fibrillation

**DOI:** 10.3390/jcm14144966

**Published:** 2025-07-14

**Authors:** Jae-Sung Choi, Jong-Ho Park

**Affiliations:** 1 Department of Thoracic and Cardiovascular Surgery, SMG-SNU Boramae Medical Center, Seoul National University College of Medicine, Seoul 07061, Republic of Korea; turejsreal@gmail.com; 2 Department of Neurology, Eunpyeong St. Mary’s Hospital, The Catholic University of Korea, 1021, Tongil Ro, Eunpyeong-gu, Seoul 03312, Republic of Korea

**Keywords:** atrial fibrillation, CHA_2_DS_2_-VASc, stroke, functional outcome

## Abstract

**Background/Objective:** Atrial fibrillation (AF) is associated with more grave and fatal outcomes than the other stroke etiologies. Left ventricular diastolic dysfunction (LVDD) is prevalent in elderly people and is associated with AF risk. We investigated whether higher preadmission CHA_2_DS_2_-VASc score is related to LVDD severity and functional outcome among stroke patients with nonvalvular AF. **Methods:** A retrospective cross-sectional analysis of data on consecutive acute ischemic stroke patients with AF within a week of onset was conducted from March 2015 to February 2018. Patients were compared by median LVDD value (13.0). CHA_2_DS_2_-VASc was assessed by score, with three categories (low risk [a CHA_2_DS_2_-VASc score of 0–2], moderate risk [3,4], and high risk [≥5]), and its individual component. Functional outcome was measured with the modified Rankin Scale (mRS) at 3 months poststroke, and unfavorable outcome was defined as mRS ≥ 3. **Results:** A total of 256 patients (mean age, 73.3 ± 10.2; male, 51.6%) were included. In multivariable regression analysis, CHA_2_DS_2_-VASc was associated with LVDD (OR 1.70, 95% CI: 1.31–2.21 for score and 9.92, 2.99–32.88 for high risk ≥ 5 versus low risk 0–2). Increasing CHA_2_DS_2_-VASc score and high risk ≥ 5 versus low risk 0–2 was associated with mRS ≥ 3 (1.72, 1.27–2.33 and 6.48, 1.37–30.60, respectively). The C-statistic of the CHA_2_DS_2_-VASc score was 0.75 (0.70–0.80) for LVDD and 0.80 (0.75–0.85) for mRS ≥ 3. The sensitivity of the CHA_2_DS_2_-VASc score for mRS ≥ 3 was higher than for LVDD. **Conclusions:** Higher preadmission CHA_2_DS_2_-VASc score can be a cumulative determinant of short-term functional outcome more than LVDD severity among stroke patients with nonvalvular AF.

## 1. Introduction

Atrial fibrillation (AF) is associated with approximately a fivefold increased risk of ischemic stroke [1], and AF-related stroke is associated with more grave and fatal outcomes than other stroke subtypes [2].

The CHA_2_DS_2_-VASc score (congestive heart failure, hypertension, age ≥ 75 years, diabetes mellitus, previous stroke/transient ischemic attack (TIA)/thromboembolism, vascular disease, age 65–74 years, and sex category) is a well-established tool for the risk stratification of stroke in patients with AF [3]. Given that the components of the CHA_2_DS_2_-VASc score share cardiovascular risk factors for atherosclerosis, a high CHA_2_DS_2_-VASc score is likely to reflect more comorbidities affecting future prognosis. For instance, a high CHA_2_DS_2_-VASc score was associated with increased risks of death, stroke recurrence, and major cardiovascular events in acute stroke patients with non-AF [4] and with stroke severity on admission and short-term functional outcome in acute stroke patients with AF [5]. However, the mechanisms linking the clinical risk factors in the CHA_2_DS_2_-VASc score to unfavorable outcomes after stroke remain unclear.

Left ventricular diastolic dysfunction (LVDD) is prevalent among elderly people [6] and is a predictor of developing nonvalvular AF [7]. A high CHA_2_DS_2_-VASc score is associated with severity of LVDD [8], which may be attributed to LV hypertrophy, the crucial risk factors of which are the components of CHA_2_DS_2_-VASc: female sex, old age, hypertension, and diabetes mellitus [9,10]. As LVDD progresses, subsequent left atrial (LA) enlargement and dysfunction may develop and contribute to thrombus formation in the LA appendage [11,12], resulting in embolic stroke in individuals with a high CHA_2_DS_2_-VASc score.

This study aimed to determine the relationships of CHA_2_DS_2_-VASc with LVDD severity and short-term functional outcome among ischemic stroke patients with nonvalvular AF.

## 2. Materials and Methods

### 2.1. Study Patients

A retrospective cross-sectional analysis of data on consecutive acute ischemic stroke patients with nonvalvular AF within a week of onset was conducted. The data were based on real-world patients admitted to a single academic center who underwent brain MRI and MR angiography (MRA) from March 2015 to February 2018. Patients were excluded if they had missing data for echocardiographic and laboratory tests; underwent incomplete or no MR imaging; had conditions affecting E/e’ including moderate to severe mitral valve disease, adult congenital heart disease, or acute coronary syndrome [13]; whose prestroke mRS was ≥3; or were lost follow-up after discharge. All methods were performed in accordance with the Declaration of Helsinki and the relevant guidelines.

### 2.2. Clinical Data

Data on demographics, clinical characteristics, laboratory findings and brain MR imaging, and medication use including antithrombotic, antihypertensive medication, and statin were retrieved. Baseline National Institutes of Health Stroke Scale (NIHSS) score on arrival and 3-month functional outcome were accessed for all patients. Functional outcome at 3 months was measured by the modified Rankin Scale (mRS). mRS defines seven clinically discrete disability categories comprising six levels of disability and one for death [14]. AF-related stroke was defined as ischemic pattern showing acute multiple territorial lesions or a single large cortical and subcortical lesion on a diffusion-weighted image [15,16]. Blood samples for fasting concentrations were drawn in the morning after an overnight fast for >8 h. The presence of AF was confirmed by electrocardiography and transthoracic echocardiography for all study patients, and additional 24 h Holter and/or transesophageal echocardiography was performed at the discretion of the responsible physician. Coronary arterial occlusive disease was defined as a history of coronary heart disease, cardiologist-diagnosed myocardial infarction, or angina pectoris, or by 3D cardiac computed tomography according to the risk assessment by a cardiologist.

### 2.3. Echocardiographic Evaluation

Transthoracic echocardiography was performed using an ultrasound system (Vivid 7, GE Healthcare, Waukesha, WI, USA) equipped with a 3.0 MHz monoplane transthoracic probe. Measurements were averaged for 5 cardiac cycles. Doppler tissue imaging was performed in parasternal and apical views. The peak early diastolic filling velocity (E) was divided by the early diastolic mitral annulus velocity (e’) measured by Doppler tissue imaging at the medial mitral annulus for E/e’ calculation. In addition, LA diameter, LV end-diastolic dimension, LV ejection fraction, and LV mass index were measured. Details of the echocardiographic methods were the same as the previous study [8].

### 2.4. CHA_2_DS_2_-VASc Score

CHA_2_DS_2_-VASc scores were calculated by assigning one or two points for each of the following conditions: history of congestive heart failure (1), age 65 to 75 (1), age ≥ 75 (2), hypertension (1), diabetes mellitus (1), prior ischemic stroke/transient ischemic attack (TIA)/thromboembolism (2), vascular disease (1), or female sex (1). Diastolic heart failure was also included as a congestive heart failure in the component of CHA_2_DS_2_-VASc if E/e′ exceeds 15 [17], since a CHA_2_DS_2_-VASc score using different echocardiographic criteria for congestive heart failure defined as E/e’ ≥ 11 was associated with the risk of thromboembolic event including stroke [18]. For the component of prior ischemic stroke/TIA/thromboembolism, we did not include the index stroke for calculating CHA_2_DS_2_-VASc scores.

### 2.5. Predictor Measure

Since the median value of E/e’ was 13 in this study, LVDD was defined as E/e’ > 13. E/e’ ≤ 13 was set as the reference for the purpose of comparison. Given that unfavorable functional outcome could be defined as mRS ≥ 3 [19] and mRS 0–2 is more associated with a favorable outcome than mRS 3–6 [20,21], study patients were dichotomized into those with mRS 0–2 and those with mRS 3–6. mRS was measured face-to-face or assessed through telephone interviews by a trained research coordinator under the help of stroke neurologists.

### 2.6. Statistical Analysis

Data are summarized as mean ± standard deviation (SD) or number of subjects (percentage), as appropriate. Comparisons across the groups were examined using the χ^2^ test or Fisher’s exact test for categorical variables and Student’s *t* test for continuous variables. E/e’ was compared according to the CHA_2_DS_2_-VASc score and category using an ANOVA, followed by the Tukey’s tau-b multiple comparisons for continuous variables, appropriately.

The CHA_2_DS_2_-VASc score is classified into low risk = 0, intermediate risk = 1, and high risk = ≥2 by the European Society of Cardiology guidelines for AF [22]. However, we categorized the scores into three groups as 0–2, 3–4, and ≥5 due to the small number of patients with each CHA_2_DS_2_-VASc score from 0 to 8 (n = 11, 24, 26, 43, 38, 55, 33, 20, and 6, respectively). The group with a CHA_2_DS_2_-VASc score of 0–2 was set as the reference group for the purposes of comparison. Bonferroni’s comparisons with the reference in association with 3-month mRS were performed. Estimated odd ratios (ORs) were calculated with reference to a CHA_2_DS_2_-VASc score of 0–2. Multivariable logistic regression analyses were performed to estimate the association of CHA_2_DS_2_-VASc with the LVDD and 3-month mRS in the following ways: (1) after adjusting for baseline covariates (AF-related stroke, smoking, baseline neurological severity, white matter hyperintensities, prestroke antihypertensive use, prestroke antithrombotic use, LA volume index, and LV ejection fraction (*p* < 0.10 in univariate analyses in Table 1)) with CHA_2_DS_2_-VASc score as model I; (2) after adjusting for baseline covariates with CHA_2_DS_2_-VASc category as model II; and (3) after adjusting for baseline covariates with CHA_2_DS_2_-VASc components. Results are given as ORs for LVDD (E/e’ > 13) and mRS ≥ 3 at 3 months with 95% confidence intervals (CIs). All analyses were conducted using IBM SPSS version 23.0 (IBM Corp., Armonk, NY, USA) and MedCalc software (version 22.016, Mariakerke, Belgium). A two-sided *p* < 0.05 was considered as the minimum level of statistical significance.

## 3. Results

### 3.1. Patient Characteristics According to Median E/e’ Value

**Figure 1:**
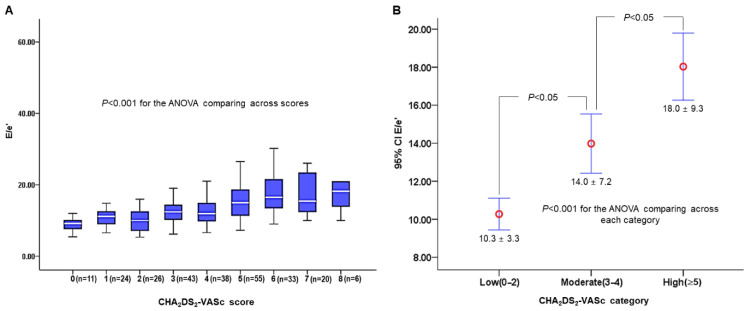
E/e’ levels according to CHA_2_DS_2_-VASc score (**A**) and category (**B**). Higher CHA_2_DS_2_-VASc score is associated with E/e’ severity (*p* < 0.001) (**A**), and E/e’ levels are significantly different across three CHA_2_DS_2_-VASc categories (*p* < 0.001) and between each CHA_2_DS_2_-VASc category by Tukey’s tau-b multiple comparisons (*p* < 0.05) (**B**).

Of the 338 stroke patients with AF, after excluding 82 patients (24.2%), a total of 256 patients (mean age, 73.3 ± 10.2; male, 51.6%) were included in this study (Supplementary Figure S1). Of 256 patients, 132 were men (51.6%), the mean age was 73.3 ± 10.2 years, and the mean CHA_2_DS_2_-VASc score was 4.0 ± 2.0 (median, 4; interquartile range, 3–5). Baseline demographic and clinical characteristics by median E/e’ value are provided in Table 1. Patients with LVDD (E/e’ > 13) were older; had higher levels of baseline stroke severity, CHA_2_DS_2_-VASc score, and LA volume index; had higher burden of white matter hyperintensities (WMH); and had greater frequencies of antihypertensive use and antithrombotic use (antiplatelets dominantly, followed by anticoagulants); meanwhile, the frequencies of smoking and LV ejection fraction were lower. Of seven CHA_2_DS_2_-VASc components, the frequencies of age ≥ 65 years, female sex, congestive heart failure, and hypertension were more likely to be greater among patients with E/e’ > 13. Regarding the index stroke etiology, AF-related stroke was more likely to be higher in patients with LVDD than in the reference group. Figure 1 depicts E/e’ levels according to each CHA_2_DS_2_-VASc score (A) and category (B). The 95% CIs of CHA_2_DS_2_-VASc score between the lowest and higher groups overlapped due to a wide range of 95% CI (A), but there were significant differences between each CHA_2_DS_2_-VASc category (low versus moderate and moderate versus high) (B). The distribution of patients’ number (percentage) by functional disability was 67 (26.2%) for mRS 0, 45 (17.6%) for mRS 1, 30 (11.7%) for mRS 2, 14 (5.5%) for mRS 3, 20 (7.8%) for mRS 4, 51 (19.9%) for mRS 5, and 29 (11.3%) for mRS 6 (Supplementary Figure S2).

### 3.2. Stroke Distribution and Stroke Outcome

**Figure 2:**
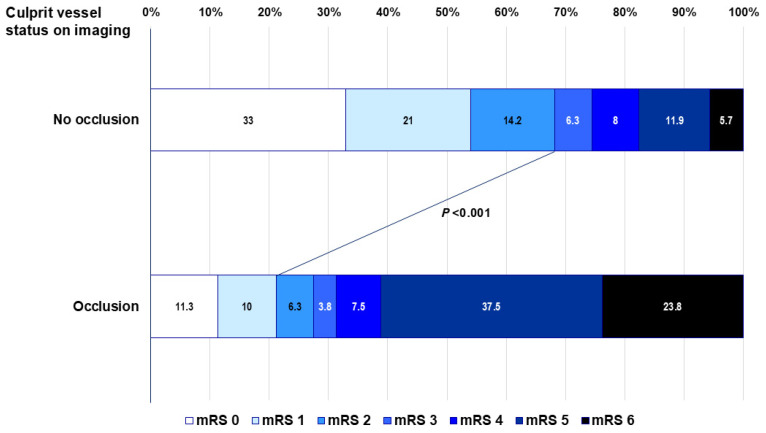
Distribution of functional outcomes by mRS at 3 months according to the involved vessel status (occlusion versus no occlusion). The proportion of mRS 3–6 was higher in patients with and without vessel occlusion (72.5% versus 31.8%, *p* < 0.001).

Occlusion of the involved vessel supplying the ischemic territory was present in 80/256 patients (31.3%). Among stroke patients with vessel occlusion, the anterior circulation was affected in 92.5% of cases, while posterior circulation and both anterior and posterior circulation were 5.0% and 2.5%, respectively (Supplementary Figure S3). Overall, 116 patients (45.3%) had an unfavorable functional outcome (mRS score 3–6) at 90 days. The proportion of patients with an mRS of 3–6 was 72.5% and 31.8% in patients with and without vessel occlusion on angiography, respectively (*p* < 0.001, Figure 2). Mortality (mRS 6) at 3 months was more frequent in patients with occlusion compared to patients without occlusion on angiography (23.8% versus 5.7%, Figure 2).

### 3.3. Associations of CHA_2_DS_2_-VASc with Predictor Outcomes

#### 3.3.1. LVDD

Results of the unadjusted and adjusted associations between CHA_2_DS_2_-VASc and LVDD are provided in Table 2. The CHA_2_DS_2_-VASc score was significantly linked to odds of LVDD (OR 1.69; 95% CI, 1.44–2.00). When referenced to CHA_2_DS_2_-VASc category 0–2, CHA_2_DS_2_-VASc categories 3–4 and ≥5 were significantly associated with increased odds of LVDD (OR 3.79; 95% CI, 1.70–8.48 and OR 12.84; 95% CI, 5.82–28.33, respectively). For CHA_2_DS_2_-VASc component, age ≥65 years (OR 6.39; 95% CI, 2.96–13.78), female sex (OR 2.77; 95% CI, 1.67–4.60), and hypertension (OR 2.29; 95% CI, 1.22–4.30) were significantly associated with increased odds of LVDD, but not for diabetes mellitus, prior stroke/TIA/thromboembolism, and vascular disease. The adjusted OR of CHA_2_DS_2_-VASc score (model I) for LVDD remained consistent (1.70; 95% CI, 1.31–2.21). For CHA_2_DS_2_-VASc category (model II), the adjusted ORs for LVDD versus CHA_2_DS_2_-VASc 0–2 were 2.51 (95% CI, 0.81–7.78) for CHA_2_DS_2_-VASc 3–4 and 9.92 (95% CI, 2.99–32.88) for CHA_2_DS_2_-VASc ≥ 5. For CHA_2_DS_2_-VASc component (model III), female sex only was independently associated with 2.82-fold odds of LVDD (2.82; 95% CI, 1.26–6.31). Among covariates included in the multivariable-adjusted models, AF-related stroke (versus non-AF-related stroke), high LA volume index, and low LV ejection fraction were significantly associated with increased odds of LVDD.

#### 3.3.2. Short-Term Functional Outcome

**Figure 3:**
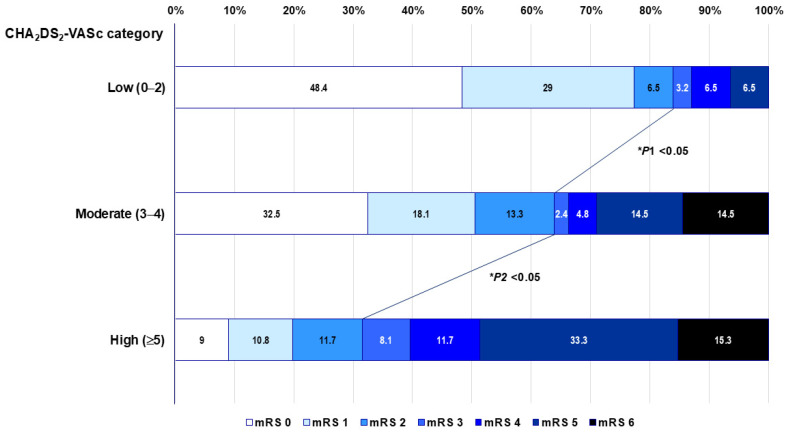
Distribution of functional outcome by mRS at 3 months according to CHA_2_DS_2_-VASc category. The oblique lines indicate statistical differences between CHA_2_DS_2_-VASc 0–2 and CHA_2_DS_2_-VASc 3–4 (P1) and CHA_2_DS_2_-VASc 3–4 and CHA_2_DS_2_-VASc ≥ 5 (P2) for mRS 3–6 versus mRS 0–2. * By chi-square test with Bonferroni adjustment. mRS, modified Rankin scale; AF, atrial fibrillation.

Results of the unadjusted and adjusted associations of CHA_2_DS_2_-VASc with short-term functional outcome are shown in Table 3. The CHA_2_DS_2_-VASc score was significantly linked to 1.8-fold odds of mRS ≥ 3 at 3 months (1.76; 95% CI, 1.49–2.07). Compared with CHA_2_DS_2_-VASc category 0–2, CHA_2_DS_2_-VASc categories 3–4 and ≥5 were significantly associated with increased odds of mRS ≥ 3 (2.94; 95% CI, 1.31–6.63 and 11.29; 95% CI, 5.14–24.79, respectively). For CHA_2_DS_2_-VASc component, age ≥ 65 years (2.56; 95% CI, 1.07–6.16), female sex (3.42; 95% CI, 1.87–6.26), hypertension (2.22; 95% CI, 1.02–4.85), diabetes mellitus (2.28; 95% CI, 1.25–4.15), and prior stroke/TIA/thromboembolism (2.46; 95% CI, 1.26–4.81) were significantly associated with increased odds of mRS ≥ 3 at 3 months. The adjusted OR of CHA_2_DS_2_-VASc score (model I) for 3-month mRS ≥ 3 remained significant (1.72; 95% CI, 1.27–2.33). For CHA_2_DS_2_-VASc category (model II), the adjusted OR for mRS ≥ 3 when referenced to CHA_2_DS_2_-VASc 0–2 was 1.64 (95% CI, 0.35–7.65) for CHA_2_DS_2_-VASc 3–4 and 6.48 (95% CI, 1.37–30.60) for CHA_2_DS_2_-VASc ≥ 5. For CHA_2_DS_2_-VASc component (model III), hypertension only was independently associated with over nine fold odds of unfavorable outcome (9.61; 95% CI, 1.97–46.96). Among covariates included in the multivariable-adjusted models, prestroke mRS, baseline stroke severity, and WMH severity were significantly associated with increased odds of unfavorable outcome, but the statistical power of LVDD was attenuated and lost significance. Figure 3 provides the distribution of functional outcome according to mRS at 3 months after stroke, which shows a greater rate of harboring unfavorable outcomes with increasing CHA_2_DS_2_-VASc category (16.1%, 33.7%, and 65.8%, respectively; *p* < 0.001). Furthermore, the frequency of mRS ≥ 3 was significantly different between each CHA_2_DS_2_-VASc category by Bonferroni adjustment (*p* < 0.05).

#### 3.3.3. Discrimination of Unfavorable Functional Outcome by CHA_2_DS_2_-VASc Risk

C-statistics of the CHA_2_DS_2_-VASc for predictor outcomes are given in Table 4, and the receiver operating characteristic curve for unfavorable outcome (mRS ≥ 3) is displayed in Supplementary Figure S4. For the CHA_2_DS_2_-VASc score, the c-statistic for LVDD and mRS ≥ 3 was 0.75 (0.70–0.80) and 0.80 (0.75–0.85), respectively. For CHA_2_DS_2_-VASc category, the c-statistic for LVDD and mRS ≥ 3 was 0.74 (0.68–0.79) and 0.77 (0.71–0.82), respectively. The sensitivity for mRS ≥ 3 was higher for CHA_2_DS_2_-VASc score than for CHA_2_DS_2_-VASc category (86.8 versus 68.4). The sensitivity for LVDD was low for both CHA_2_DS_2_-VASc score and CHA_2_DS_2_-VASc category.

## 4. Discussion

In this analysis of 256 stroke patients with nonvalvular AF, we observed that higher CHA_2_DS_2_-VASc score was significantly associated with greater burden of LVDD and unfavorable stroke outcome at 3 months. The odds of LVDD showed a stepwise increase with a higher CHA_2_DS_2_-VASc category and were substantially increased by nearly 10-fold for the highest category (≥5). The odds of mRS ≥ 3 at 3 months increased with a higher CHA_2_DS_2_-VASc category and were significantly increased over 6-fold for the highest category.

CHA_2_DS_2_-VASc was not primarily designed to reflect LVDD or functional outcome but rather to help guide the decision to estimate stroke risk in AF patients and to consider anticoagulants in men with a score of 1 or more and women with a score of 2 or more [22]. Nevertheless, c-statistic values of CHA_2_DS_2_-VASc for the odds of LVDD and unfavorable functional outcome in our study were all over 0.70 for both score and category, suggesting modest value for prediction [23].

A previous study investigated the influence of LVDD on the LA appendage, showing that E/e′ ≥ 13 was an independent predictor of LA appendage thrombus (OR 3.5) due to blood stasis or impaired LA appendage function [11]. The E/e’ level 13 is same as the median value in our study. However, despite the relation of LVDD to LA thrombogenesis [11,12] and consequent grave embolic stroke, our study did not show an independent association between LVDD and 3-month functional outcome. This lack of power may not be too surprising because functional outcome following AF-related stroke depends on the involved vessel status and distribution: vessel occlusion of anterior circulation at the time of stroke or contemporary revascularization therapy was independently associated with an mRS of 3–6 at 90 days [24], the findings of which may outweigh the impact of LVDD. As such, in our study, vessel occlusion was seen in 80/256 patients (31.3%) with the anterior circulation dominance (92.5%). As shown in Figure 2, the proportion of patients with mRS ≥ 3 was 72.5% and 31.8% in patients with and without vessel occlusion, respectively. Our findings are in accordance with a recent study that the presence of thrombus in LA/LA appendage was more related to relevant vessel occlusion and associated with higher odds of mRS ≥ 3 at 3 months among stroke patients with nonvalvular AF [25]. We could not, however, investigate the correlation with the functional outcome due to unavailability of the data on LA/LA appendage thrombus.

Additionally, the difference of E/e’ values between patients with and without vessel occlusion was not significant (15.5 ± 7.8 versus 14.5 ± 8.3, Supplementary Figure S5). Another plausible explanation is the presence of variables affecting the tissue Doppler index E/e’ levels: technical factors, physiological factors including beat-to-beat variability in AF, left bundle branch block, older age, female sex, and clinical factors (e.g., diabetes mellitus, LV hypertrophy, reduced LV ejection fraction, and moderate to severe aortic stenosis), all of which can increase E/e’ [26]. In our study, patients with E/e’ > 13 were older, had higher frequencies of female and congestive heart failure, and had lower levels of LV ejection fraction than those with E/e’ ≤ 13, the findings of which might have attenuated the independent power of LVDD on functional outcome. Likewise, a previous systemic review reported that E/e’ was limited in reflecting LVDD even in heart failure with preserved ejection fraction, suggesting a less accurate diagnostic measure [13]. In this regard, CHA_2_DS_2_-VASc seems to provide a greater predictive value for functional outcome, which is reflected in the sensitivity of CHA_2_DS_2_-VASc score versus LVDD.

Among CHA_2_DS_2_-VASc components in our study, female sex was independently associated with increased odds of LVDD. Age and male sex are associated with increased risk of LVDD [27]. Our contradictive finding is due to older age in women than men (75.8 ± 9.3 versus 71.0 ± 10.4, *p* < 0.001). As age [28], female sex [29], diabetes mellitus [30], and stroke severity [31] are known to be associated with unfavorable outcome after stroke, our study showed that age, female sex, and diabetes tended to be related to mRS ≥ 3. Those nonsignificant associations might be ascribed to the fact that the study patients were old (median age 74) and harbored many of CHA_2_DS_2_-VASc components (median score 4). The baseline stroke severity by NIHSS was higher in women than men (9.4 ± 6.6 versus 6.6 ± 5.9, *p* < 0.001).

Prestroke antithrombotic medications were largely antiplatelet drugs (e.g., aspirin, clopidogrel, or sarpogrelate) that did not contribute to the prevention of cardioembolic stroke. Our findings need to be validated through a large prospective study to evaluate functional outcome according to CHA_2_DS_2_-VASc in AF patients who are on non-vitamin K antagonist medications.

### Limitations

Our study still has several limitations. First, this is a retrospective analysis of consecutively collected data on stroke patients with nonvalvular AF in a single academic center. Thus, our findings cannot provide a causal relationship between CHA_2_DS_2_-VASc and functional outcome poststroke and LVDD and need careful interpretation to generalize to overall stroke survivors with nonvalvular AF. Second, the number of study patients was small in each CHA_2_DS_2_-VASc score, thereby leading to wide confidence intervals, affecting the precision of estimates for predictor outcomes. Third, we could not correlate CHA_2_DS_2_-VASc with the presence of LAA/LAA appendage thrombus in association with functional outcome due to unavailability of the data on transesophageal echocardiography or multi-detector coronary computed tomography. Indeed, it was practically difficult for elderly patients with comorbid medical conditions or severe neurological deficits to implement that study. Fourth, there were a lot of study patients who had influencing factors on E/e’ levels. The majority of stroke patients with AF are older and have comorbid conditions such as vascular risk factors and cardiac dysfunctions, but our patients might reflect a real-world setting. Lastly, despite adjusting for numerous covariates in multivariable models, there may remain unmeasured confounders, such as underlying sarcopenia or socioeconomic status reflecting attention to health care, which might have influenced the functional outcome.

## 5. Conclusions

This study suggests that preadmission CHA_2_DS_2_-VASc score may exert as a cumulative prediction marker for short-term functional outcome and reflect the burden of LVDD, whereas the baseline LVDD was not related to 3-month stroke outcome. To enable clinicians to predict prognosis and establish preventive and management strategies in high-risk individuals with nonvalvular AF, more data on CHA_2_DS_2_-VASc score in association with LA/LAA appendage thrombus and functional outcomes with large-scale stroke patients with nonvalvular AF need to be validated.

## Figures and Tables

**Table 1:** Baseline characteristics of 256 ischemic stroke patients with AF according to diastolic function as E/e’.

	Dichotomization by Median Value		*p* Value
	E/e’ ≤ 13 (*n* = 132)	E/e’ > 13 (*n* = 124)	
Age, years	70.6 ± 11.1	76.2 ± 8.1	<0.001
BMI, kg/m^2^	23.5 ± 3.3	24.0 ± 4.1	0.264
Onset time to arrival, h	19.1 ± 32.6	17.2 ± 33.7	0.657
Prestroke mRS	0.44 ± 1.10	0.69 ± 1.29	0.103
Baseline NIHSS score	6.9 ± 6.2	9.1 ± 6.4	0.007
AF type			0.191
Persistent	111 (84.1)	112 (90.3)	
Paroxysmal	21 (15.9)	12 (9.7)	
CHA_2_DS_2_-VASc score *	3.1 ± 1.9	4.9 ± 1.6	<0.001
CHA_2_DS_2_-VASc components *			
Age ≥ 65 years	88 (66.7)	115 (92.7)	<0.001
Female sex	48 (36.4)	76 (61.3)	<0.001
Congestive heart failure	6 (4.6)	90 (72.6)	<0.001
Hypertension	95 (72.0)	106 (85.5)	0.010
Diabetes mellitus	40 (30.3)	48 (38.7)	0.188
Prior stroke/TIA/thromboembolism *	29 (22.0)	36 (29.0)	0.200
Vascular disease †	26 (19.7)	22 (17.7)	0.750
Coronary arterial occlusive disease	18 (13.6)	11 (8.9)	0.244
Smoking	35 (26.5)	15 (12.1)	0.004
HbA1c, %	6.2 ± 1.2	6.3 ± 1.4	0.409
Total cholesterol, mg/dL	164.4 ± 40.0	164.4 ± 37.4	0.998
Triglycerides, mg/dL	100.7 ± 58.3	96.8 ± 58.0	0.598
HDL-C, mg/dL	42.3 ± 11.8	42.6 ± 11.9	0.823
LDL-C, mg/dL	101.7 ± 34.4	101.3 ± 34.3	0.923
NT Pro-BNP, pg/mL	2414.8 ± 7625.2	4978.0 ± 19,478.8	0.341
Homocysteine, µmol/L	14.3 ± 6.9	14.0 ± 6.1	0.727
Uric acid, mg/dL	6.0 ± 1.9	6.1 ± 1.8	0.631
Arterial occlusion at baseline	38 (28.8)	42 (33.9)	0.229
IV ± IA or IA only treatment	29 (22.0)	27 (21.8)	1.000
Prestroke antihypertensive use	60 (59.4)	61 (77.2)	0.016
Prestroke antithrombotic use	53 (40.2)	65 (52.4)	0.060
Prestroke statin use	29 (22.0)	33 (26.6)	0.466
Echocardiographic data			
LA volume index, mL/m^2^	43.8 ± 16.4	57.4 ± 23.1	<0.001
LV mass index, g/m^2^	109.6 ± 83.6	114.5 ± 33.7	0.640
LV ejection fraction (%)	60.7 ± 9.6	57.6 ± 11.1	0.017
Index stroke etiology			0.011
AF-related	118 (89.4)	121 (97.6)	
Non-AF-related	14 (10.6)	3 (2.4)	
WMH by Fazekas score			<0.001
0	22 (16.7)	6 (4.8)	
1	51 (38.6)	34 (27.4)	
2	52 (39.4)	65 (52.4)	
3	7 (5.3)	19 (15.3)	
Cerebral atherosclerosis (≥50%), n			
0	101 (76.5)	91 (73.4)	0.499
1	19 (14.4)	16 (12.9)	
2 or more	12 (9.1)	17 (13.7)	

Values provided are number (%) or mean ± SD, as appropriate, unless otherwise stated. AF, atrial fibrillation; BMI, body mass index; NIHSS, National Institutes of Health Stroke Scale; mRS, modified Rankin Scale; INR, International Normalized Ratio; TIA, transient ischemic attack; LDL-C, lower-density lipoprotein cholesterol; IA, intraarterial; IV, intravenous; LA, left atrial; LV, left ventricular. * Index vascular event not counted. † Includes prior myocardial infarction, peripheral artery disease, aortic plaque, or cerebral atherosclerotic stenosis (≥50%).

**Table 2:** Unadjusted and adjusted ORs of CHA_2_DS_2_-VASc for LVDD (E/e’ > 13).

	Unadjusted OR (95% CI)	*p* Value	Model I	*p* Value	Model II	*p* Value	Model III	*p* Value
			Adjusted OR (95% CI)		Adjusted OR (95% CI)		Adjusted OR (95% CI)	
CHA_2_DS_2_-VASc score *	1.69 (1.44–2.00)	<0.001	1.70 (1.31–2.21)	<0.001	…	…	…	…
CHA_2_DS_2_-VASc category *								
0–2	Referent				Referent		…	
3–4	3.79 (1.70–8.48)	0.001	…	…	2.51 (0.81–7.78)	0.110	…	…
≥5	12.84 (5.82–28.33)	<0.001	…	…	9.92 (2.99–32.88)	<0.001	…	…
CHA_2_DS_2_-VASc component								
Age ≥ 65 years	6.39 (2.96–13.78)	<0.001	…	…	…	…	2.56 (0.83–7.90)	0.101
Female sex	2.77 (1.67‒4.60)	<0.001	…	…	…		2.82 (1.26‒6.31)	0.012
Hypertension	2.29 (1.22–4.30)	0.010	…	…	…	…	1.45 (0.51–4.10)	0.484
Diabetes mellitus	1.45 (0.87–2.44)	0.158	…	…	…	…	1.27 (0.57–2.84)	0.564
Prior stroke/TIA/thromboembolism *	1.45 (0.83–2.56)	0.196	…	…	…	…	1.14 (0.47–2.75)	0.775
Vascular disease	0.88 (0.47–1.65)	0.689	…	…	…	…	1.31 (0.43–3.99)	0.638
AF-related stroke	4.79 (1.34–17.08)	0.016	17.46 (2.08–146.38)	0.008	5.22 (1.27–21.56)	0.022	11.32 (1.28–100.24)	0.029
Smoking	0.38 (0.20–0.74)	0.004	1.06 (0.32–3.51)	0.925	0.38 (0.09–1.56)	0.181	1.08 (0.32–3.69)	0.901
Prestroke mRS	1.19 (0.96–1.47)	0.105	…	…	2.04 (1.10–3.79)	0.023	…	…
Baseline stroke severity †	1.06 (1.02–1.10)	0.008	1.00 (0.94–1.06)	0.938	1.22 (1.12–1.32)	<0.001	1.01 (0.95–1.07)	0.746
WMH by Fazekas score	2.05 (1.47–2.86)	<0.001	1.25 (0.73–2.14)	0.419	2.29 (1.14–4.58)	0.020	1.43 (0.84–2.44)	0.185
LA volume index, mL/m^2^	1.04 (1.02–1.06)	<0.001	1.03 (1.01–1.05)	0.007	1.03 (1.01–1.05)	0.007	1.03 (1.01–1.05)	0.006
LV ejection fraction, %	0.97 (0.95–1.00)	0.021	0.96 (0.91–1.00)	0.067	0.95 (0.91–1.00)	0.044	0.94 (0.90–0.99)	0.020
Prestroke antihypertensive use	0.43 (0.22–0.84)	0.012	0.90 (0.37–2.20)	0.816	0.80 (0.33–1.96)	0.628	0.80 (0.32–2.03)	0.640
Prestroke antithrombotic use	0.61 (0.37–1.00)	0.050	1.40 (0.63–3.10)	0.415	1.29 (0.58–2.86)	0.530	1.29 (0.57–2.91)	0.546

LVDD, left ventricular diastolic dysfunction; E, early transmitral diastolic peak velocity; e’, mitral annular diastolic peak velocity; TIA, transient ischemic attack; AF, atrial fibrillation; mRS, modified Rankin Scale; WMH, white matter hyperintensities; LA, left atrium; OR, odds ratio; CI, confidence interval. * Index stroke or transient ischemic attack not calculated. † National Institutes of Health Stroke Scale.

**Table 3:** Unadjusted and adjusted ORs of CHA_2_DS_2_-VASc for unfavorable functional outcome (mRS 3–6) at 3 months.

	Unadjusted OR (95% CI)	*p* Value	Model I	*p* Value	Model II	*p* Value	Model III	*p* Value
			Adjusted OR (95% CI)		Adjusted OR (95% CI)		Adjusted OR (95% CI)	
CHA_2_DS_2_-VASc score *	1.76 (1.49–2.07)	<0.001	1.72 (1.27–2.33)	<0.001	…	…	…	…
CHA_2_DS_2_-VASc category *								
0–2	Referent		…		Referent		…	
3–4	2.94 (1.31–6.63)	0.009	…	…	1.64 (0.35–7.65)	0.528	…	…
≥5	11.29 (5.14–24.79)	<0.001	…	…	6.48 (1.37–30.60)	0.018	…	…
CHA_2_DS_2_-VASc component								
Congestive heart failure	1.66 (0.92–2.99)	0.091	…	…	…	…	0.92 (0.27–3.20)	0.898
Age ≥ 65 years	2.56 (1.07–6.16)	0.035	…	…	…	…	2.07 (0.45–9.64)	0.353
Female sex	3.42 (1.87‒6.26)	<0.001	…	…	…	…	2.05 (0.75‒5.63)	0.164
Hypertension	2.22 (1.02–4.85)	0.045	…	…	…	…	9.61 (1.97–46.96)	0.005
Diabetes mellitus	2.28 (1.25–4.15)	0.007	…	…	…	…	2.52 (0.96–6.64)	0.060
Prior stroke/TIA/thromboembolism *	2.46 (1.26–4.81)	0.008	…	…	…	…	2.08 (0.74–5.85)	0.167
Vascular disease	0.88 (0.42–1.84)	0.741	…	…	…	…	1.85 (0.58–5.88)	0.297
AF-related stroke	0.54 (0.20–1.47)	0.226	…	…	…	…	…	…
Smoking	0.37 (0.18–0.73)	0.004	0.53 (0.14–2.02)	0.349	0.54 (0.15–2.02)	0.361	0.42 (0.10–1.79)	0.238
Prestroke mRS	3.49 (2.23–5.46)	<0.001	1.92 (1.04–3.55)	0.037	2.10 (1.13–3.93)	0.020	2.39 (1.19–4.78)	0.014
Baseline stroke severity †	1.23 (1.16–1.30)	<0.001	1.20 (1.11–1.29)	<0.001	1.19 (1.10–1.28)	<0.001	1.21 (1.12–1.32)	<0.001
WMH by Fazekas score	2.63 (1.84–3.78)	<0.001	2.27 (1.17–4.39)	0.015	2.37 (1.20–4.66)	0.013	1.97 (0.98–4.00)	0.059
E/e’	1.09 (1.04–1.13)	<0.001	1.02 (0.97–1.08)	0.403	1.03 (0.97–1.09)	0.369	1.05 (0.97–1.14)	0.241
LA volume index, mL/m^2^	1.01 (1.00–1.02)	0.218	…	…	…	…	…	…
LV ejection fraction, %	0.99 (0.96–1.01)	0.204	…	…	…	…	…	…
Prestroke antihypertensive use	0.40 (0.20–0.80)	0.010	1.05 (0.38–2.89)	0.925	0.83 (0.31–2.24)	0.715	1.58 (0.52–4.80)	0.421
Prestroke antithrombotic use	0.97 (0.59–1.59)	0.909	…	…	…	…	…	…

mRS, modified Rankin Scale; TIA transient ischemic attack; AF, atrial fibrillation; WMH, white matter hyperintensities; E, early transmitral diastolic peak velocity; e’, mitral annular diastolic peak velocity; LA, left atrium; LV, left ventricle; OR, odds ratio; CI, confidence interval. * Index stroke or transient ischemic attack not calculated. † National Institutes of Health Stroke Scale.

**Table 4:** Discrimination of LVDD and unfavorable functional outcome (mRS 3–6) at 3 months by CHA_2_DS_2_-VASc risk.

CHA_2_DS_2_-VASc score	LVDD		mRS 3–6	
C-statistic (95% CI)	OR 0.75, 95% CI: 0.70–0.80	*p* < 0.001	OR 0.80, 95% CI: 0.75–0.85	*p* < 0.001
Sensitivity	66.1		86.8	
Specificity	75.8		62.7	
CHA_2_DS_2_-VASc category (0–2, 3–4, ≥5)	LVDD		mRS 3–6	
C-statistic (95% CI)	OR 0.74, 95% CI: 0.68–0.79	*p* < 0.001	OR 0.77, 95% CI: 0.71–0.82	*p* < 0.001
Sensitivity	63.7		68.4	
Specificity	75.8		76.7	

LVDD, left ventricular diastolic dysfunction; mRS, modified Rankin scale; OR, odds ratio; CI, confidence interval.

## Data Availability

The anonymized data used in this study are available from the corresponding author upon reasonable request.

## Supplementary Materials

The following supporting information can be downloaded at: https://www.mdpi.com/article/10.3390/jcm14144966/s1. Figure S1. Flowchart of patient inclusion. AF, atrial fibrillation. Figure S2. Distribution of functional disability according to mRS. mRS, modified Rankin scale. The values provided are percentages. Figure S3. Distribution of vessel occlusion stroke according to location. Figure S4. Discrimination of unfavorable functional outcome (mRS 3-6) at 90 days by CHA2DS2-VASc risk. mRS, modified Rankin scale. Figure S5. LVDD level between patients with and without vessel occlusion. LVDD indicates left ventricular diastolic dysfunction defined as E/e’ > 13.
