# Supplementary material for: Preadmission CHA2DS2-VASc Scores on Diastolic Function and Functional Outcome After Stroke with Nonvalvular Atrial Fibrillation
Source: J Clin Med. 2025 Jul 14;14(14):4966. doi: 10.3390/jcm14144966 (PMC12295445; doi:10.3390/jcm14144966)
Supplement: Supplementary file 1 [file jcm-14-04966-s001.zip › jcm-3669260-supplementary.pdf]

SUPPLEMENTARY MATERIALS

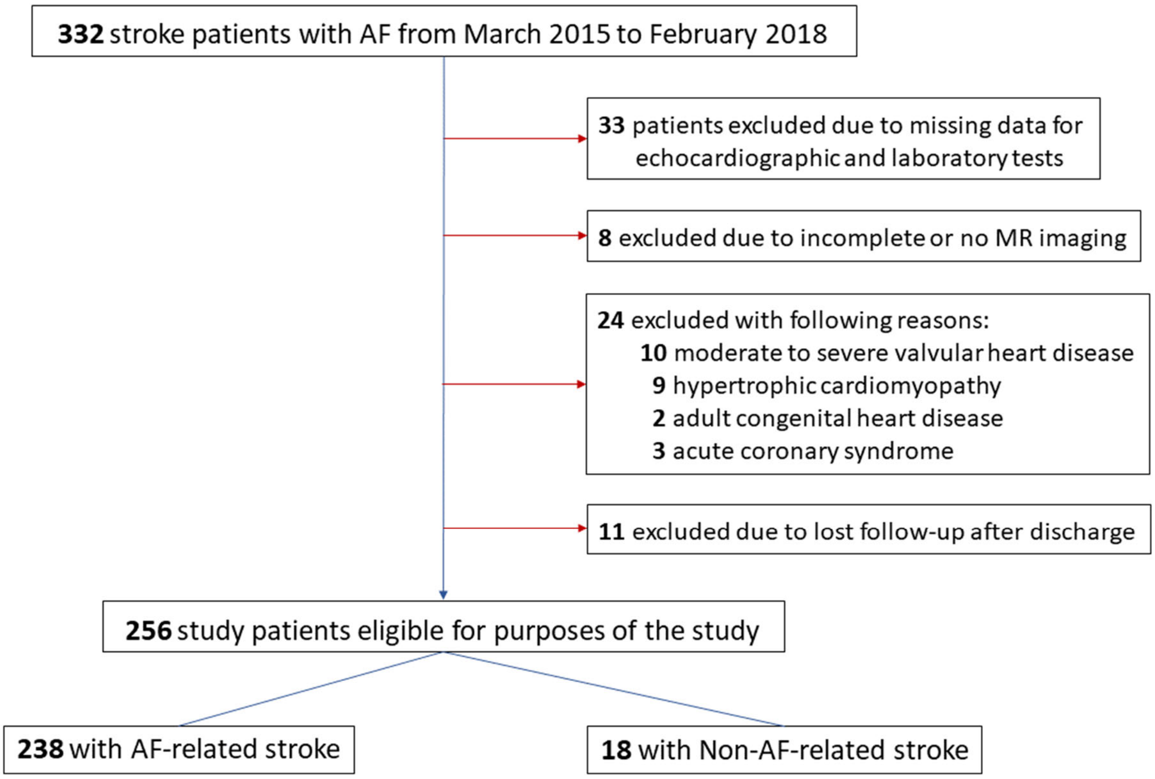

Supplementary Figure S1. Flowchart of patient inclusion. AF, atrial fibrillation.

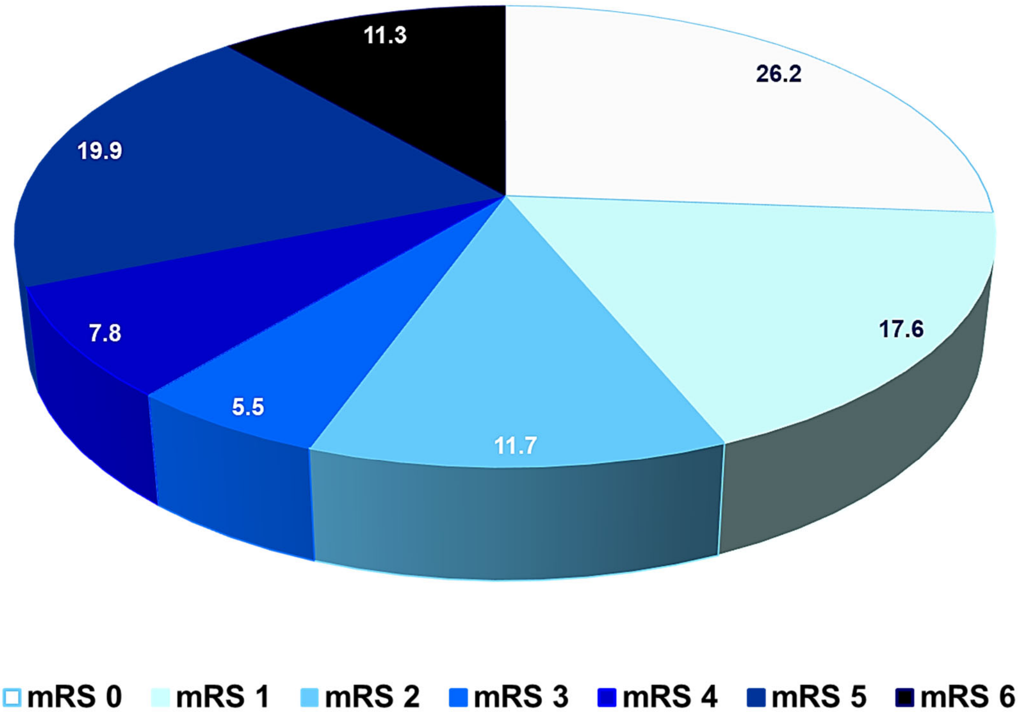

Supplementary Figure S2. Distribution of functional disability according to mRS. mRS, modified Rankin scale. The values provided are percentage.

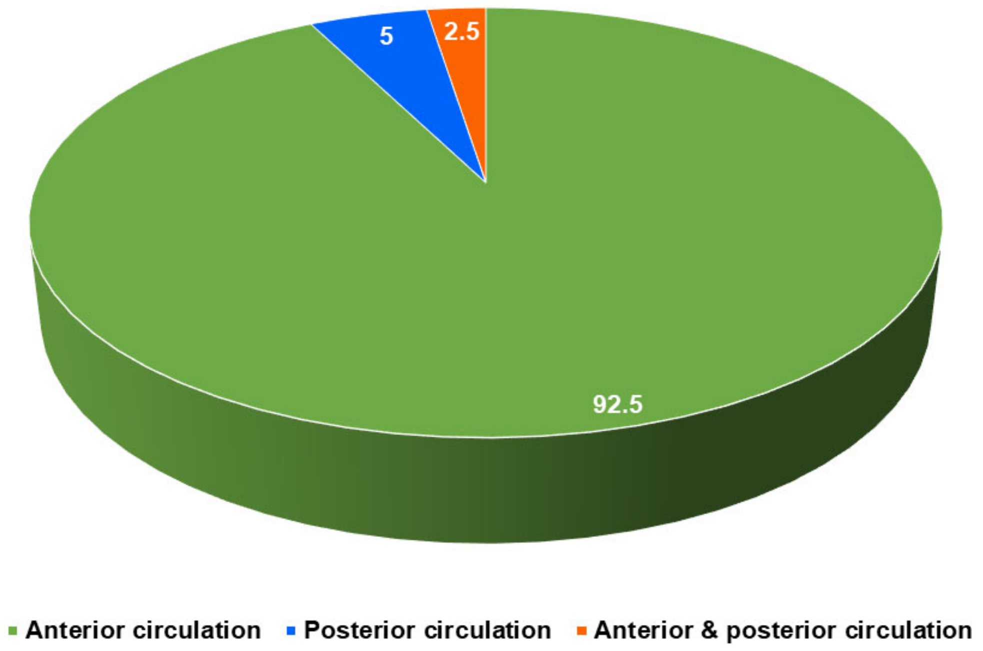

Supplementary Figure S3. Distribution of vessel occlusion stroke according to location.

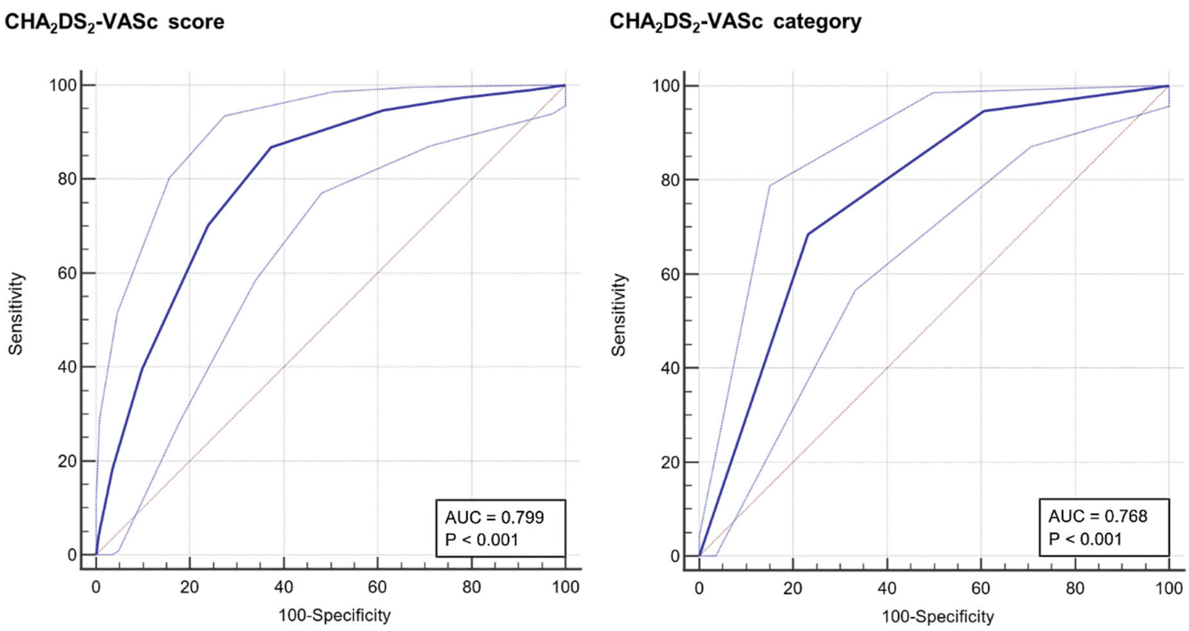

Supplementary Figure S4. Discrimination of unfavorable functional outcome (mRS 3-6) at 90 days by CHA_2DS_2-VASc Risk. mRS, modified Rankin scale.

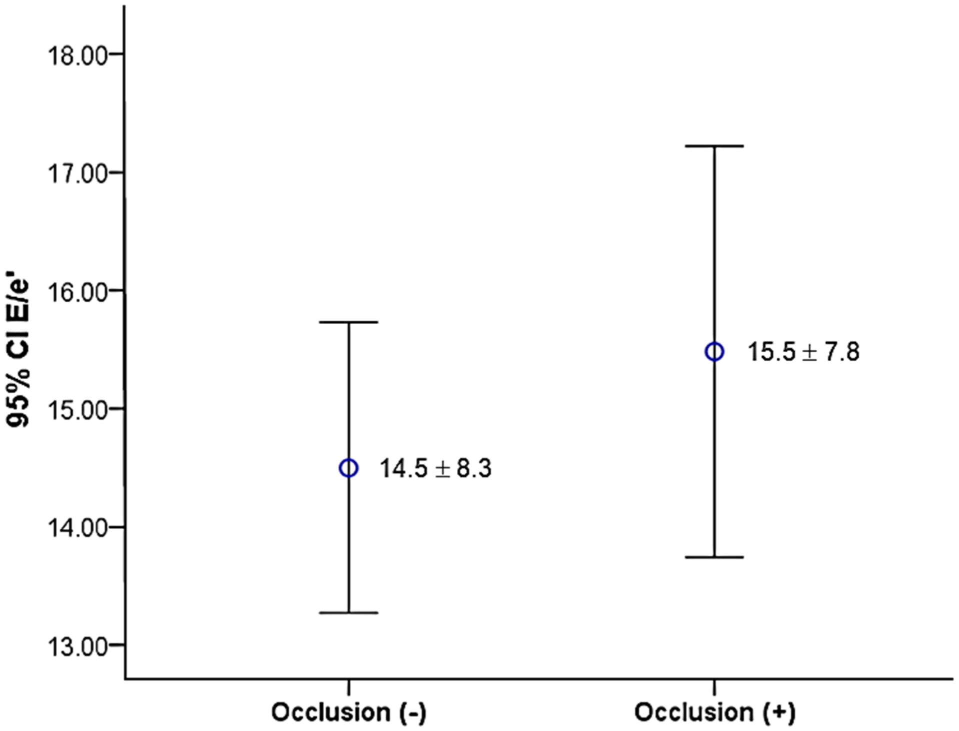

Supplementary Figure S5. LVDD level between patients with and without vessel occlusion. LVDD indicates left ventricular diastolic dysfunction defined as E/e’ >13.
